# Alterations in Rat Hippocampal Glutamatergic System Properties after Prolonged Febrile Seizures

**DOI:** 10.3390/ijms242316875

**Published:** 2023-11-28

**Authors:** Alexandra V. Griflyuk, Tatyana Y. Postnikova, Sergey L. Malkin, Aleksey V. Zaitsev

**Affiliations:** Sechenov Institute of Evolutionary Physiology and Biochemistry of RAS, 44, Toreza Prospekt, Saint Petersburg 194223, Russia; griflyuk.al@mail.ru (A.V.G.); tapost2@mail.ru (T.Y.P.); adresatt@gmail.com (S.L.M.)

**Keywords:** febrile seizures, hyperthermia, hippocampus, maximal electroshock seizure threshold test, epilepsy, local field potential

## Abstract

Febrile seizures during early childhood may result in central nervous system developmental disorders. However, the specific mechanisms behind the impact of febrile seizures on the developing brain are not well understood. To address this gap in knowledge, we employed a hyperthermic model of febrile seizures in 10-day-old rats and tracked their development over two months. Our objective was to determine the degree to which the properties of the hippocampal glutamatergic system are modified. We analyzed whether pyramidal glutamatergic neurons in the hippocampus die after febrile seizures. Our findings indicate that there is a reduction in the number of neurons in various regions of the hippocampus in the first two days after seizures. The CA1 field showed the greatest susceptibility, and the reduction in the number of neurons in post-FS rats in this area appeared to be long-lasting. Electrophysiological studies indicate that febrile seizures cause a reduction in glutamatergic transmission, leading to decreased local field potential amplitude. This impairment could be attributable to diminished glutamate release probability as evidenced by decreases in the frequency of miniature excitatory postsynaptic currents and increases in the paired-pulse ratio of synaptic responses. We also found higher threshold current causing hind limb extension in the maximal electroshock seizure threshold test of rats 2 months after febrile seizures compared to the control animals. Our research suggests that febrile seizures can impair glutamatergic transmission, which may protect against future seizures.

## 1. Introduction

Febrile seizures are the most common type of seizures in childhood, with a prevalence rate of 2–5% among children aged 6 months to 5 years [1,2,3]. Febrile seizures can be classified as simple or complex, depending on the duration and recurrence of episodes. Simple seizures typically last less than 15 min and occur no more than once a day. About 70% of all reported cases of childhood seizures are simple seizures. Complex seizures are defined as seizures that last longer than 15 min or involve repeated episodes over a 24-h period. The most severe form of complex febrile seizures is febrile status epilepticus, which involves seizures lasting more than 30 min [4]. The relationship between febrile seizures and the development of temporal lobe epilepsy is uncertain. However, cohort studies indicate that children with complex febrile seizures have a significantly higher risk of developing temporal lobe epilepsy than those with simple febrile seizures [5,6,7].

To understand the mechanisms underlying potential epileptogenesis after febrile seizures, animal models must replicate the basic features of such seizures in children, including age and body temperature at onset. In this study, we utilized a well-established model of febrile seizures [8] that enabled tight control over the seizure duration, thereby facilitating accurate modeling of the complex form of febrile seizures.

Several mechanisms are thought to cause epileptogenesis after complex febrile seizures. In particular, limbic seizures in the mature brain can result in the loss of vulnerable cells in the hippocampus, which may contribute to the development of epilepsy. Febrile seizures primarily occur in early childhood when the nervous system is still developing. Whether febrile seizures cause neuronal death remains a topic of debate. Clinical cases have described hippocampal edema in children within 48 h of prolonged febrile seizures, which resolves within 5 days [9]. Studies on animal models of febrile seizures indicate that there is no significant death of hippocampal neurons, but they do indicate cell damage and mossy fiber overgrowth [10,11]. As part of this study, we evaluated the incidence of neuronal death in the rat hippocampus at varying intervals following prolonged febrile seizures to examine the short- and long-term effects of these seizures on hippocampal morphology.

Neuroinflammation may accompany febrile seizure development and epileptogenesis. Studies have revealed increased levels of pro-inflammatory cytokines in the serum of children who experienced febrile seizures [12,13] and in animal models of febrile seizures [14]. This can result in chronic hyperexcitability of the neuronal circuits and epilepsy development [15]. However, it remains unclear whether there is an increase in neuronal excitability after febrile seizures. One study shows a reduction in the amplitude of population spikes in CA1 field neurons when the Schaffer collaterals are stimulated, as a result of enhanced inhibitory postsynaptic currents in the rat hippocampus one week after febrile seizures [16]. However, when the Schaffer collaterals are stimulated with a series of stimuli, epileptiform activity develops only in slices obtained from animals after febrile seizures compared to controls, indicating the increased excitability of the hippocampal neuronal circuits [17]. Our study focused on examining excitatory synaptic transmission in the hippocampus of animals at different time points after febrile seizures to assess the acute and long-term changes. In this study, we selected three age points to examine. The first point pertains to the acute brain response to febrile seizures. The second point (P20) corresponds to early childhood in humans, while the third point (P60) corresponds to adulthood [18,19]. In addition, we assessed the seizure threshold of the animals in vivo two months post-febrile-seizure.

## 2. Results

### 2.1. Febrile Seizures Provoke Neuronal Loss in the CA1 Region of the Rat Hippocampus 

**Figure 1:**
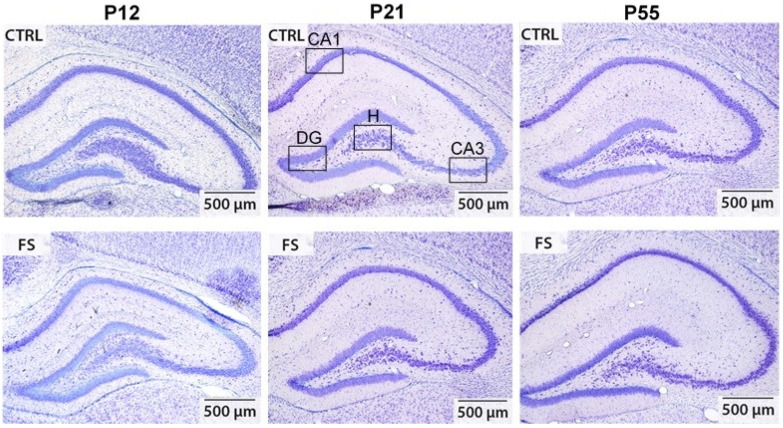
Representative images (50× magnification) show Nissl-stained, 20 μm thick frontal sections of the hippocampus in control (CTRL) and experimental post-FS (FS) rats of varying ages. The black boxes highlight the regions where neuron quantification was conducted, including CA1, CA3, hilus (H), and dentate gyrus (DG).

**Figure 2:**
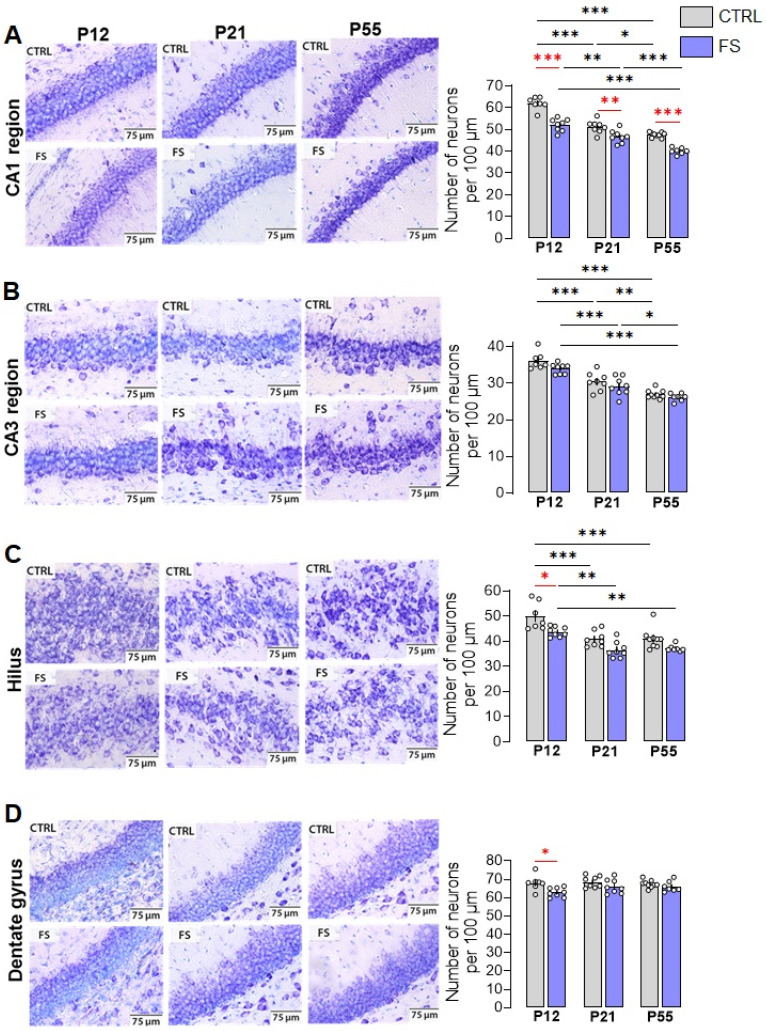
Nissl staining of neurons in hippocampal areas CA1 (**A**), CA3 (**B**), hilus (**C**), and dentate gyrus (**D**) in control (CTRL) and experimental post-FS (FS) rats. Diagrams showing the number of Nissl-stained neurons per 100 µm cell layer. The circles show the individual values for each rat. Asterisks indicate significant differences between groups according to Tukey’s post hoc test: * *p* < 0.05, ** *p* < 0.01, *** *p* < 0.001. Between-group differences are shown in red.

Whether febrile seizures induce the loss of hippocampal neurons remains a topic of controversy, with conflicting data from clinical studies and animal experiments [10,11,20,21]. To assess the effect of prolonged febrile seizures at P10 on neuronal damage in rats, we used Nissl staining on brain slices and determined the number of neurons in different areas of the hippocampus, including the CA1, CA3, hilus, and dentate gyrus regions, at different ages (P12, P21, and P55) (Figure 1 and Figure 2). 

A two-way ANOVA revealed the significant effect of febrile seizures, age and their interaction on neuron number only in the hippocampal CA1 region (Figure 2A; F(FS)_1,41_ = 101.7; *p* < 0.0001; F(Age)_2,41_ = 110.8; *p* < 0.0001; F(FS×Age)_2,41_ = 4.8; *p* < 0.01). Tukey’s post hoc tests revealed significant differences in the number of neurons both between control animals and animals after febrile seizures (post-FS rats) at different ages, and a decrease in neurons during development (P12: control: 62.1 ± 1.1 neurons per 100 µm, post-FS: 52.1 ± 1.0; P21: control: 51.2 ± 1.0, post-FS: 46.8 ± 1.0; P55: control: 47.6 ± 0.4, post-FS: 40.1 ± 0.6).

In the hippocampal CA3 region and hilus, a two-way ANOVA revealed significant effects of age and febrile seizures, but there was no interaction between the factors (Figure 2B: CA3 region: F(FS)_1,41_ = 6.7; *p* < 0.05; F(Age)_2,41_ = 75.5; *p* < 0.0001; F(FS×Age)_2,41_ = 0.5; *p* = 0.60. Figure 2C: Hilus: F(FS)_1,41_ = 21.9; *p* < 0.0001; F(Age)_2,41_ = 26.6; *p* < 0.0001; F(FS×Age)_2,41_=0.5; *p* = 0.64). In the CA3 area, Tukey’s post hoc tests revealed a decrease in the number of neurons with increasing age in both the control and experimental groups (P12: control: 36.2 ± 0.9 neurons per 100 µm, post-FS: 33.9 ± 0.5; P21: control: 30.4 ± 0.9, post-FS: 29.1 ± 0.9; P55: control: 26.9 ± 0.4, post-FS: 26.2 ± 0.4). In the hilus, in addition to age-related changes, there is a decrease in the number of neurons in animals after febrile seizures at P12 (P12: control: 49.9 ± 2.1 neurons per 100 µm, post-FS: 43.8 ± 0.7; P21: control: 40.9 ± 1.1, post-FS: 36.6 ± 1.1; P55: control: 41.1 ± 1.4, post-FS: 37.2 ± 0.5).

In the dentate gyrus, only the effect of febrile seizures was revealed (Figure 2D; F(FS)_1,41_ = 10.9; *p* < 0.01; F(Age)_2,41_ = 1.4; *p* = 0.25; F(FS×Age)_2,41_ = 1.5; *p* = 0.23). Tukey’s post hoc tests revealed a reduction in the number of neurons in animals after febrile seizures compared to the control group only at P12 (P12: control: 68.1 ± 1.5 neurons per 100 µm, post-FS: 62.9 ± 0.9; P21: control: 68.6 ± 1.1, post-FS: 66.1 ± 1.4; P55: control: 67.4 ± 0.7, post-FS: 66.1 ± 1.1).

We have shown that febrile seizures lead to a reduction in the number of neurons in different areas of the hippocampus. However, the CA1 field displayed the greatest susceptibility, with a decrease in the number of neurons in post-FS animals across all three age groups. A decrease in the number of neurons was only observed at P12 in the hilus and dentate gyrus. There were no changes related to febrile seizures in the CA3 region.

### 2.2. Synaptic Neurotransmission in the Hippocampus Changed after Febrile Seizures

**Figure 3:**
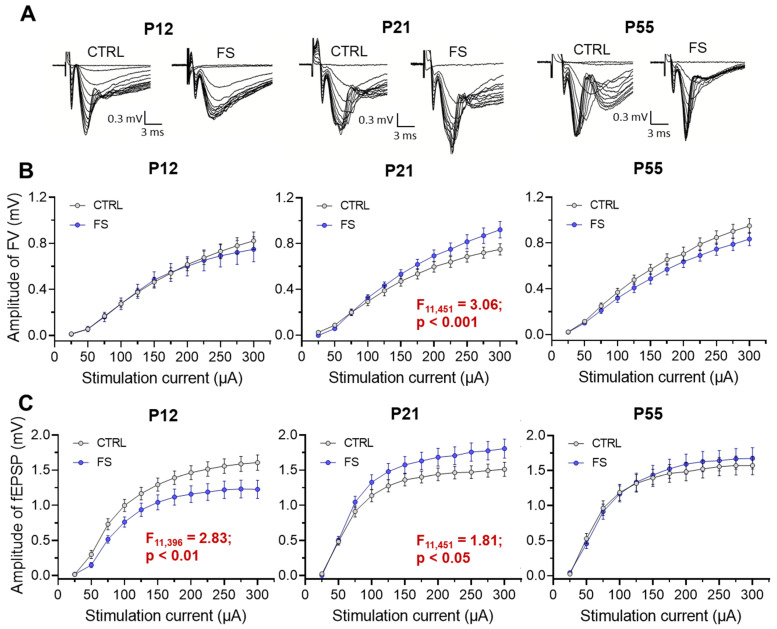
Synaptic neurotransmission in the hippocampus changed after febrile seizures. (**A**) Representative examples of fEPSPs recorded at different strengths of extracellular stimulation in the control (CTRL) and after-febrile-seizure animals (FS) of different ages (P12, P21, P55). Stimulation–response relationships for presynaptic fiber volley (FV) amplitude (**B**) and fEPSP amplitudes (**C**) recorded from the hippocampal CA1 region. Data shown as means ± standard errors of the means.

To evaluate the basic synaptic neurotransmission at the CA3–CA1 pyramidal neuron synapses in hippocampal slices, afferent fibers underwent electrical stimulation at different current intensities (25–300 mA). The synaptic neurotransmission was evaluated in both post-FS animals and control animals across different ages (P12, P21-23, P51-55; Figure 3). The fiber volley (FV) amplitude, reflecting the number of CA3 axons that evoke action potentials, and the fEPSPs amplitude, reflecting the sum of excitatory postsynaptic responses occurring in the dendrites of the CA1 pyramidal neurons in response to afferent fiber stimulation, were both recorded in each slice.

At P12 in post-FS animals, the fEPSP amplitude was reduced compared to the control (F_11,396_ = 2.83; *p* < 0.01, control: *n* = 21 slices; *N* = 10 rats; post-FS: *n* = 17 slices; *N* = 10 rats), while no changes in the FV amplitude were observed (F_11,396_ = 0.32; *p* = 0.98). Conversely, at P21, an increase in the amplitudes of both FV (F_11,451_ = 3.06; *p* < 0.001, control: *n* = 16 slices; *N* = 10 rats; post-FS: *n* = 27 slices; *N* = 12 rats) and fEPSP (F_11,451_ = 1.81; *p* < 0.05) was observed in post-FS animals. At the age of 51–55 days, no statistically significant differences in these parameters were found between the control animals and post-FS animals (amplitudes of FV: F_11,440_ = 1.31; *p* = 0.21, control: *n* = 16 slices; *N* = 11 rats; post-FS: *n* = 24 slices; *N* = 10 rats and amplitudes of fEPSP: F_11,440_ = 0.63; *p* = 0.80).

### 2.3. Short-Term Synaptic Plasticity of Hippocampal Neurons Changes in Rats Two Days after Febrile Seizures 

**Figure 4:**
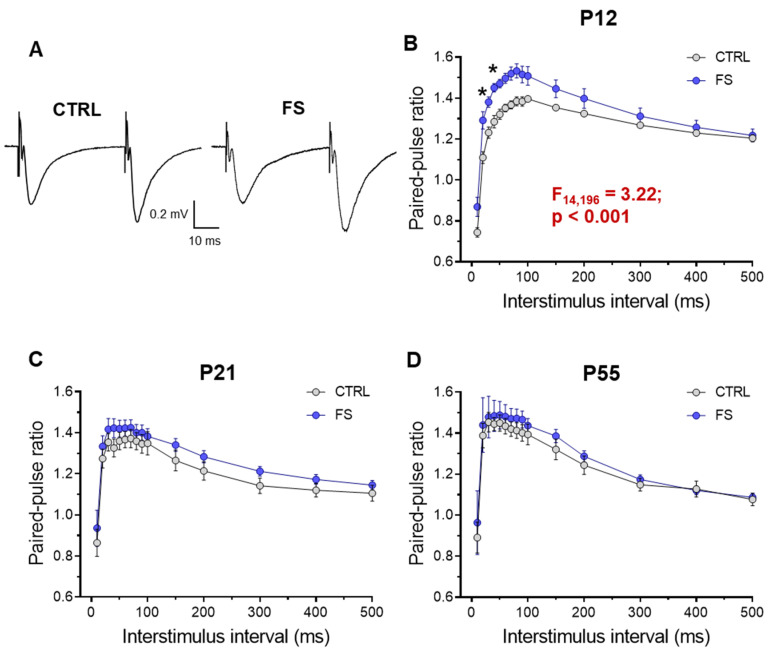
Paired-pulse facilitation altered in hippocampal slices 2 days after febrile seizures. (**A**) Representative examples of paired-pulse responses from the hippocampus in control rats (CTRL) and rats after febrile seizures (FS) at P12 using interstimulus intervals of 40 ms. (**B**–**D**) Diagrams of paired-pulse facilitation in rat hippocampal slices at P12 (**B**), P21-23 (**C**), and P51-55 (**D**) days at different interstimulus intervals. Asterisks indicate significant differences according to Tukey’s post hoc test: * *p* < 0.05.

The decrease in fEPSP apmlitude without a change in FV amplitude in rats two days after febrile seizures may be related to changes in the probability of glutamate release in the Schaffer collaterals. To assess possible changes in the probability of mediator release after febrile seizures, we assessed the short-term synaptic plasticity (STP) in rats of different ages [22]. To this end, we used a paired-pulse stimulation with an interstimulus interval of 10 to 500 ms and compared the paired-pulse ratio (PPR) at different intervals in control animals (P12: *n* = 8 slices; *N* = 6 rats; P21: *n* = 12 slices; *N* = 7 rats; P55: *n* = 9 slices; *N* = 6 rats) and post-FS animals (P12: *n* = 8 slices; *N* = 5 rats; P21: *n* = 15 slices; *N* = 8 rats; P55: *n* = 9 slices; *N* = 6 rats). Repeated-measure ANOVA revealed significant changes in the PPR only in animals two days after febrile seizures, whereas no differences for the control group were found in animals at P21 and P55 (P12: F_14,196_ = 3.22; *p* < 0.001; P21: F_14,336_ = 0.26; *p* = 0.99; P55: F_14,224_ = 0.12; *p* = 0.99; Figure 4).

This experiment shows a decrease in the probability of mediator release in the hippocampal CA3-CA1 synapses two days after febrile seizures. Later, however, these changes are no longer observed. 

### 2.4. Frequency of Miniature Excitatory Postsynaptic Current Is Reduced Two Days after Febrile Seizures

Since we observed significant changes in synaptic transmission in rats 2 days after experiencing febrile seizures, we analyzed the properties of the miniature excitatory postsynaptic currents (mEPSCs) in the CA1 neurons, including their amplitudes, kinetics, and frequency, in the control and post-FS groups, recorded at –80 mV. 

**Figure 5:**
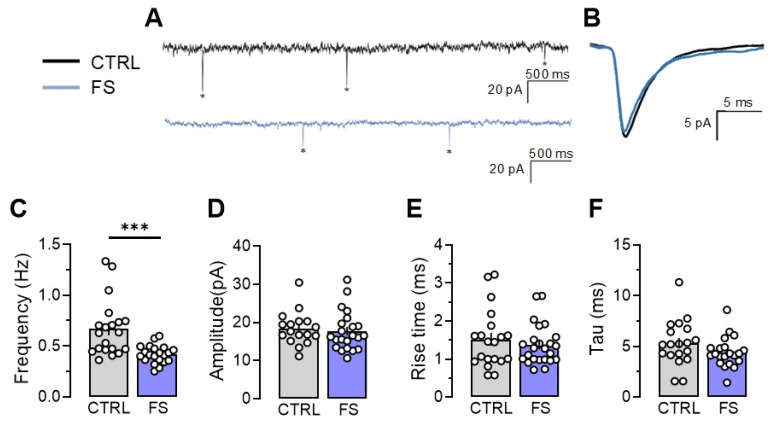
Frequency of miniature excitatory postsynaptic current (mEPSC) is reduced two days after experiencing febrile seizures. (**A**) Representative current responses from CA1 pyramidal neurons recorded at −80 mV and (**B**) examples of averaged mEPSCs in control (CTRL) and post-FS (FS) animals. The asterisks indicate individual mEPSCs. The frequency (**C**), amplitude (**D**), rise time (**E**), and decay time constant (**F**) of mEPSC in the control and post-FS groups are presented. Asterisks indicate significant differences between groups according to Student’s test: * *p* < 0.05, *** *p* < 0.001.

Our findings indicate that the frequency of the mEPSCs decreased by 47% (Control: 0.67 ± 0.06 Hz; *n* = 20 neurons; *N* = 3 rats, post-FS: 0.42 ± 0.02 Hz; *n* = 21 neurons; *N* = 4 rats, *p* < 0.001). Meanwhile, the other mEPSC parameters, including amplitude (control: 18.4 ± 1.1 pA, *n* = 17, post-FS: 17.7 ± 1.2 pA, *n* = 22, *p* = 0.66), rise time (control: 1.52 ± 0.19 ms, *n* = 20, post-FS: 1.37 ± 0.11 ms, *n* = 24, *p* = 0.46), and decay time constant (control: 5.32 ± 0.39 ms, *n* = 19, post-FS: 4.42 ± 0.32 ms, *n* = 21, *p* = 0.14), remained unaltered (Figure 5). 

This suggests that presynaptic mechanisms likely trigger changes in synaptic strength in the CA1 neurons. Consequently, our research shows that febrile seizures can result in impaired glutamatergic transmission during the first few days following a seizure, which might serve as a protective factor in reducing the possibility of future seizures.

### 2.5. Rats after Febrile Seizures Have an Increased Threshold for Maximal Electroshock Seizure

**Figure 6:**
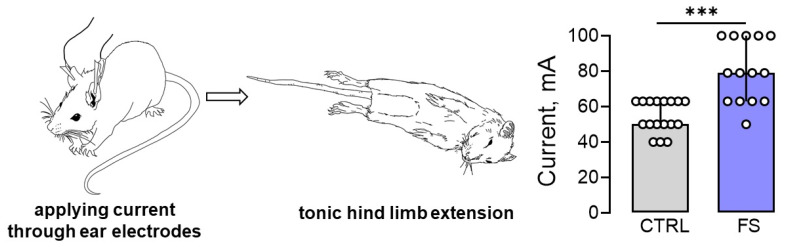
The maximal electroshock seizure threshold (MEST) increased in rats 2 months after febrile seizures. Image of a rat with tonic hind limb extension induced by applying current through ear electrodes. Diagrams illustrating the differences in threshold currents in the control (CTRL) and post-FS (FS) animals required for tonic hind limb extension. The circles show the individual values for each rat. Asterisks indicate significant differences between groups according to Mann–Whitney U test: *** *p* < 0.001. Data are represented as median with interquartile range.

Since prolonged febrile seizures in early childhood may increase the risk of developing epilepsy, it was hypothesized that the threshold for seizure onset would be reduced in post-FS rats. To evaluate this hypothesis, the maximal electroshock seizure threshold (MEST) test was conducted on rats 2 months after febrile seizures (*n* = 14) and on control animals of the same age (*n* = 17). The results showed that the post-FS rats had a significantly higher threshold for tonic hind limb extension (U_17,14_ = 27, *p* < 0.001, Figure 6).

Thus, our experimental model of febrile seizures revealed a surprising outcome: early childhood febrile seizures may actually raise the threshold for tonic seizure generation rather than lower it, as previously believed.

## 3. Discussion

Febrile seizures are the most common type of seizure in young children [4,23,24]. However, the association of febrile seizures with subsequent hippocampal damage and the development of temporal lobe epilepsy remains undetermined. A retrospective analysis of patients with temporal lobe epilepsy shows a high prevalence of febrile seizures in their history, which may suggest the etiological role of these seizures in the development of temporal lobe epilepsy [25,26]. However, according to population-based and prospective epidemiological studies, febrile seizures do not progress to temporal lobe epilepsy [27]. Most research indicates that boys are more prone to febrile seizures than girls [28,29,30]. Interestingly, some experimental studies have shown that there is a difference in sex-related susceptibility to febrile seizures [31], other seizures in early childhood [32], and neonatal hypoxia ischemia [33]. Males seem to exhibit more severe cognitive and behavioral deficits compared to females with matched conditions [31,32,33]. To exclude possible sex differences from the analysis, we used only male rats in this study, so it should be kept in mind that any conclusions drawn are specific to the male sex; it cannot be excluded that the effects of febrile seizures may be somewhat different in female rats.

In this study, we aimed to investigate whether hippocampal neuronal death and changes in hippocampal excitability occur at different developmental ages after prolonged febrile seizures in early life.

We found that the CA1 region of the hippocampus showed the most significant loss of neurons. Furthermore, two days post-seizure, there was impaired glutamatergic transmission, with a lower probability of mediator release and a decline in baseline synaptic transmission at the CA3–CA1 synapses. Interestingly, the baseline neurotransmission in rats increases at 3 weeks of age, 11 days after seizures. However, there were no alterations in baseline synaptic transmission found 40–45 days after seizures. Despite this, the threshold for developing tonic convulsions in animals was observed to have increased two months after febrile seizures in comparison to the control group.

In this study, we confirmed the widely held belief that seizures in the developing brain do not cause a large loss of neurons. Prior research has indicated that there is minimal neuronal death in the hippocampus 20 h after febrile seizures [10], whereas there is no neuronal death in adult animals that have experienced febrile seizures during their early stages of life [10,11]. Our findings generally support these previous observations.

Four hippocampal regions were compared in this study across three animal age groups. After febrile seizures, the number of neurons decreased in the CA1 region, hilus, and dentate gyrus within two days, the earliest point of the study. Conversely, there was no loss of neurons in the CA3 region of the hippocampus. Nevertheless, after 10 days and 1.5 months, only the CA1 region displayed observable differences between the post-FS and control groups. These findings are consistent with data from other immature animal seizure models (lithium–pilocarpine model of status epilepticus and kainic acid model of temporal lobe epilepsy), where the hippocampal CA1 neurons have been shown to be more vulnerable than other hippocampal regions [34,35,36]. One possible explanation for why the CA1 neurons in the hippocampus are more prone to febrile seizures at P10 is the delayed development of synaptic inhibition in the CA1 compared to CA3 region during early postnatal ontogeny [37]. It is worth mentioning that neurogenesis persists in the dentate gyrus during the postnatal period, potentially accounting for the absence of differences at later stages. Nonetheless, the dentate gyrus cells generated after febrile seizures exhibit augmented spontaneous excitatory input [38].

We observed an age-related decrease in the number of neurons in the CA1 and CA3 regions, as well as in the hilus, both in the control group and in animals after febrile seizures. The number of neurons in the hilus does not differ from the control group by 21 days of age due to this process. However, differences persist in the CA1 region because neuronal death in this area is more pronounced in the early days after seizures.

Concurrently with examining morphological changes, we investigated the excitatory synaptic transmission in the CA3–CA1 neurons of the hippocampus. The most significant changes were observed two days after febrile seizures, with a reduction in synaptic transmission efficacy, changes in short-term plasticity, and a decrease in the frequency of miniature excitatory synaptic currents. Overall, the findings indicate a decrease in the probability of mediator release in the hippocampal CA3–CA1 synapses. These changes in the probability of glutamate release may shift the balance of excitation and inhibition toward inhibition and reduce the risk of seizure activity in hippocampal neuronal networks. 

Opposite changes can occur in various models of seizures and epilepsy. For instance, in the 4-aminopyridine in vitro model, no changes were noticed in the frequency of the mEPSCs and the paired amplitude ratio of the evoked responses, one hour after epileptiform activity. However, potentiation of the synaptic transmission was observed due to postsynaptic changes [39]. Similarly, one hour after neonatal hypoxic seizures, researchers observed an increase in synaptic transmission attributed to an increased mEPSC amplitude mediated by AMPA receptors [40]. Moreover, the authors of this study found that hypoxia-induced seizures resulted in an increased mEPSC frequency, indicating a combined presynaptic and postsynaptic potentiation [40]. The amplitude, but not the frequency, of the mEPSCs recorded from the CA1 pyramidal neurons was found to be increased in the slices taken from animals with pilocarpine-induced status epilepticus [41]. In another study using a lithium–pilocarpine model, the evoked EPSC amplitudes were increased 20–60 days after the pilocarpine seizures, and then decreased further into the chronic phase of the epilepsy model [42]. In the repeated low-dose kainate model of epilepsy, 1 week after the induction of seizures, an increased mEPSC frequency was observed, although the amplitudes were similar to the control [43]. 

In many models of seizures and epilepsy, synaptic potentiation results from the NMDA-dependent incorporation of AMPA receptors, which includes an increase in the proportion of calcium-permeable AMPA receptors [40,41,44,45,46]. Neuroinflammation can increase the proportion of calcium-permeable AMPA receptors. Specifically, administering bacterial lipopolysaccharide in experimental studies showed elevated GluA1 expression following lipopolysaccharide treatment in two-week-old [47] and two-month-old rats [48]. Conversely, febrile seizures cause a rapid decrease in the proportion of calcium-permeable AMPA receptors in the synapses of pyramidal neurons [49]. 

The difference may be due to the febrile seizure model used in our experiments, which does not lead to the development of chronic epilepsy in rats. Therefore, the synaptic changes rapidly disappear after the seizures since no epileptogenesis occurs. Nonetheless, in a comparable febrile seizure model, it was revealed that the glutamate release probability increased two months following the seizure [38]. It should be noted, however, that these modifications were identified in the dentate granule cells instead of the CA1 region.

Unexpectedly, we found a higher current threshold for the development of tonic seizures in in vivo experiments. However, previous studies have shown an increased susceptibility to seizures in adult rats at least three months after febrile seizures [17] and electroencephalographically recorded epileptiform discharges in the limbic system [50]. In contrast, a previous study found that young animals exhibited reduced susceptibility to pentylenetetrazole-induced seizures 20 days after experiencing febrile seizures [51]. These results are consistent with our own findings in animals observed 2 months after seizures. It is possible that the discrepancies in results are due to differences in the age of the animals evaluated for seizure susceptibility. It is possible that after febrile seizures, neural circuit excitability may be reduced in animals as a compensatory measures to reduce the risk of developing recurrent seizures. Nevertheless, further research is necessary to support this hypothesis.

However, reducing the excitatory transmission in the immature brain could potentially delay its further development. This is because the maturation of and morphological changes in astrocytes rely on neuronal activity, and astrocytes regulate synaptogenesis in the immature brain [52,53]. This could hamper the maturation of synaptic plasticity processes, leading to potential cognitive impairment, as previously demonstrated [54].

## 4. Materials and Methods

### 4.1. Animals

**Figure 7:**
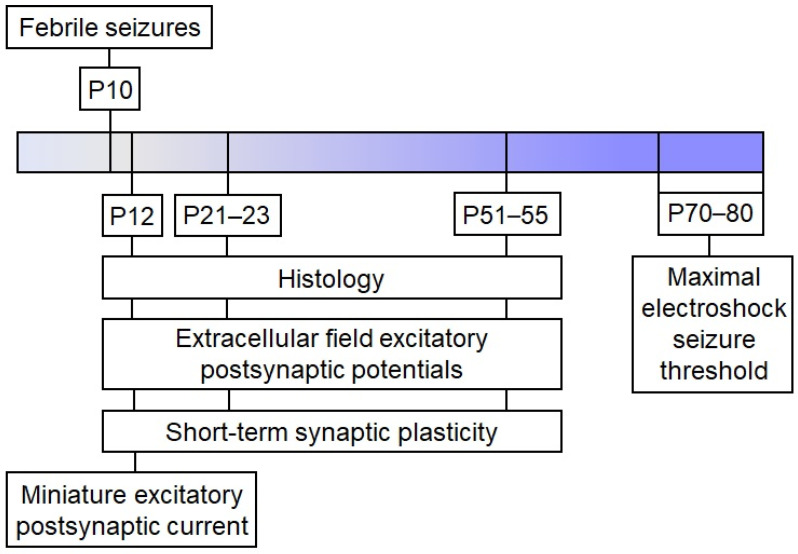
The experimental design.

Male Wistar rats were utilized in this study. Animals were housed in standard conditions with unrestricted access to food and water. The Ethics Committee of the Sechenov Institute of Evolutionary Physiology and Biochemistry approved all experiments and ensured compliance with local guidelines for laboratory animal welfare. These conditions fully comply with international regulations for animal experimentation. The sequence of experiments performed is shown in the scheme (Figure 7).

### 4.2. Febrile Seizure Model

Febrile seizures were induced on postnatal day 10 as described previously [8,54]. Briefly, the P10 pups were placed at the bottom of a glass chamber for 30 min and exposed to a regulated stream of heated air, keeping the chamber temperature at 46 °C. Most animals had their body temperature rise to 39 °C within the first 10 min under these conditions, and often showed facial automatisms, sometimes accompanied by unilateral body flexion. This was followed by myoclonic twitching of the hind limbs, followed by clonic convulsions. Rectal body temperature was measured every two minutes and maintained below 41 °C during episodes of convulsions. The study included a total of 63 animals with FS durations of at least 15 min. After hyperthermia, the pups were placed on a cold surface until their core temperature was normalized, and then returned to their nest. The littermates utilized as controls were removed from the nest for the same duration but were kept at room temperature (N = 65).

### 4.3. Histology

At P12, P21–23, and P51–55, the rats were anesthetized with a mixture of Zoletil (3 mg per 100 g) and xylazine (50 µL per 100 g) diluted in saline. Afterward, the rats were perfused transcardially with phosphate-buffered saline (PBS, pH 7.4, 0.01 M), followed by 4% paraformaldehyde (PFA) in PBS. Subsequently, the animals were decapitated, and the brain removed and fixed in 4% PFA at 4 °C for at least 2 days. After fixation, the brains were cryoprotected in 30% sucrose. The brains were frozen in cooled (<−50 °C) isopentane (78-78-4, Isopentane Solution, Sigma-Aldrich, St. Louis, MO, USA) and stored at −80 °C. 

Serial 20 μm thick frontal sections were cut on a Bright OTF5000 cryostat (Bright Instrument Co., Ltd., Huntingdon, UK) at −2.6 to −3.6 mm bregma, placed on Super Frost Plus-coated slides (J1800AMNZ, Fisher Scientific UK Ltd., Loughborough, UK), and air-dried for 1 day. Nissl staining was performed as previously described in detail [55]. The Nissl-stained sections were analyzed using a Leica AF7000 microscope (Leica Microsystems, Wetzlar, Germany) at ×400 magnification. Neuronal counts were conducted on every fifth section, resulting in a yield of 6–8 sections from a single rat hippocampus. The analyzed sections were spaced 100 µm apart. The ImageJ 1.52a software (U.S. National Institutes of Health, Bethesda, MD, USA) was used to calculate the number of neurons per 100 μm in the CA1, CA3, hilus, and dentate gyrus cell layers from digital micrographs.

### 4.4. Brain Slice Preparation

At P12, P21-23, and P51-55, the rats were decapitated and their brains were quickly removed. Using an HM 650 V vibratome (Microm, Walldorf, Germany), horizontal brain slices (400 μm) were cut in chilled artificial cerebrospinal fluid (ACSF) at a temperature of 0 °C. The ACSF contained 126 mM NaCl, 24 mM NaHCO_3_, 2.5 mM KCl, 2 mM CaCl_2_, 1.25 mM NaH_2_PO_4_, 1 mM MgSO_4_, and 10 mM glucose and was aerated with carbogen (95% O_2_ and 5% CO_2_). Afterward, the slices were allowed to recover for 1 h at 35 °C in oxygenated ACSF. 

### 4.5. Field Potential Recordings

Field potentials were recorded in the CA1 stratum radiatum of the hippocampus using glass microelectrodes (0.2–1.0 MΩ), following the procedures outlined in previous studies [56]. Each slice was stimulated with increasing amplitude currents (25 to 300 μA, 25 μA increments) to measure the fEPSP and FV amplitudes. Paired pulses with varying interstimulus intervals were administered every 20 s to ascertain the paired-pulse ratio (PPR), calculated as the amplitude ratio between the first fEPSP and the second. The intervals ranged from 10 to 500 ms.

### 4.6. Patch-Clamp Recordings 

The recordings were performed at 30 °C. The neurons in the pyramidal layer of the CA1 hippocampus were visualized using a Zeiss Axioskop 2 microscope (Zeiss, Oberkochen, Germany) equipped with differential interference contrast optics and a video camera (Sanyo VCB-3512P, Sanyo Electric, Osaka, Japan). Patch electrodes with a resistance of 2-4 MΩ were fabricated from borosilicate glass capillaries (Sutter Instrument, Novato, CA, USA) using a P-1000 micropipette puller (Sutter Instrument). For recording the miniature excitatory synaptic currents (mEPSCs), we employed a solution based on potassium gluconate. The solution’s composition in mM was as follows: 114 K-gluconate, 6 KCl, 0.2 EGTA, 10 HEPES, 4 ATP-Mg, and 0.3 GTP. The pH level was adjusted to 7.25 using KOH.

Signals were recorded using a MultiClamp 700B patch-clamp amplifier (Molecular Devices, Sunnyvale, CA, USA), an InstruTECH LIH 8 + 8 ADC/DAC module (HEKA, Stuttgart, Germany), and the WinWCP 5.2.7 software (University of Strathclyde, Glasgow, UK). The data underwent 3 kHz filtering and 16.67 kHz sampling. The access resistance was less than 20 MΩ for all whole-cell recordings included in the sample and remained stable (≤20% increase) across the experiments. No series resistance compensation was utilized during the experiment.

The mEPSC recordings were conducted at a membrane potential of −70 mV in the presence of 0.5 μM TTX (Alomone Labs, Jerusalem, Israel). The mEPSCs were detected offline using the Clampfit 10.0 software (Molecular Devices) and their characteristics were analyzed utilizing software based on open-source SciPy and NumPy libraries for the Python programming language (Python Software Foundation, Wilmington, DE, USA).

### 4.7. Maximal Electroshock Seizure Threshold (MEST)

To evaluate the susceptibility to seizures in animals, we measured the MEST two months following febrile seizures. Current was applied via ear electrodes using an ECT Unit 57800 pulse generator (Ugo Basile, Gemonio, Italy), using stimulation currents ranging from 12 to 100 mA with log scale intervals of 0.1. The pulse frequency was set at 150 Hz, pulse duration at 0.8 s, and pulse width at 0.9 ms. We determined the minimum current necessary to observe tonic hind limb extension for each animal. On day one, the animal received a current of 40 mA. If tonic hind limb extension was not observed, the current was increased by 1 step. If tonic hind limb extension occurred at 40 milliamperes, the current was decreased by 1 step. The tests were repeated every 2–3 days.

### 4.8. Statistical Analysis

Statistical analysis was conducted using the Statistica 8.0 (Systat Software, Inc., Palo Alto, CA, USA) and GraphPad Prism 8 software (GraphPad Software, San Diego, CA, USA). We identified outliers using Dixon’s Q-test and tested for normal distribution using the Kolmogorov–Smirnov test. We used Student’s test, two-way ANOVA, or repeated-measure ANOVA as appropriate to assess the statistical significance, followed by Tukey’s post hoc test, as described in the text. Statistical analysis of the MEST test data was conducted using the Mann–Whitney U test. Results were presented as mean ± standard error of the mean for normal distribution or median and interquartile range for non-normal distribution. A *p*-value less than 0.05 was considered statistically significant.

## Data Availability

The data presented in this study are available on request from the corresponding author.
